# Effect of respiratory rehabilitation training on elderly patients with COVID-19: A protocol for systematic review and meta-analysis

**DOI:** 10.1097/MD.0000000000022109

**Published:** 2020-09-11

**Authors:** Huan Yan, Yonghong Ouyang, Lang Wang, Xiangjun Luo, Qian Zhan

**Affiliations:** a Department of Acupuncture; b Department of General Medicine, The First Affiliated Hospital of Hunan Traditional Chinese Medical College; c Department of Metabolic Endocrinology, The Affiliated ZhuZhou Hospital XiangYa Medical College CSU, Zhuzhou, Hunan 412000; d Department of Pneumology, West China Hospital, Sichuan University, Chengdu, Sichun 610041, China.

**Keywords:** COVID-19, elderly, meta-analysis, protocol, respiratory rehabilitation

## Abstract

**Background:**

Patients with the Corona Virus Disease 2019 (COVID-19) often see their respiratory, physical, and psychological functions impaired to varying degrees, especially for the elderly patients. Timely respiratory rehabilitation intervention for such patients may improve their prognoses. However, its relative effectiveness has not been proved. Therefore, this study is purposed to determine the effect of respiratory rehabilitation on elderly patients with COVID-19.

**Methods:**

This study will search the following electronic databases: Embase, MEDLINE, PubMed, Cochrane Library, China national knowledge infrastructure database, Wan Fang database, Chinese Science and Technology Periodical Database, and Chinese Biomedical Literature Database, with the retrieval period running from their inception to August 2020. All randomized controlled trials of respiratory rehabilitation training on elderly patients with COVID-19 are collected, and the data are selected and extracted independently according to the pre-designed inclusion/exclusion criteria. Cochrane bias risk assessment tool is used to evaluate the method quality and bias risk. All data analyses will be implemented by using Revman5.3 and Stata14 software.

**Results:**

This study will make a high-quality and comprehensive evaluation of the efficacy of respiratory rehabilitation training on elderly patients with COVID-19.

**Conclusion:**

The conclusions of this systematic review will deliver more convincing evidence.

**Ethics and dissemination:**

The private information collected from individuals will not be published. And this systematic review will also not involve impairing the participants’ rights. Ethical approval is not required. The results may be published in a peer-reviewed journal or disseminated in relevant conferences.

## 1 Introduction

In December 2019, the Corona Virus Disease 2019 COVID-19) was broken out in Wuhan, China. Because of its strong infectivity, it had since spread widely over a very short period of time.^[1,2]^ Patients with COVID-19 have varying degrees of impairments in their respiratory, physiological, and psychological functions, especially for elderly patients.^[3]^ According to the experience of condition-improving patients and discharged patients, timely respiratory rehabilitation intervention can improve the prognosis, maximize the preservation of functions, and enhance the quality of life.^[4–6]^

As an important non-drug intervention in the treatment of respiratory diseases, respiratory rehabilitation training is increasingly accepted in clinical practice,^[7–9]^ which mainly includes sports training (such as breathing training, aerobic training, and resistance training) as well as disease health education and self-management.^[10–12]^ Among them, exercise training, as one of the most effective rehabilitation methods for respiratory diseases, forms the core part. And a large batch of clinical and basic studies have demonstrated that respiratory rehabilitation training, with exercise training as the main component, can improve the activity ability and quality of life of patients with respiratory diseases by strengthening their lung functions, reducing the airway resistance, improving immune functions, and boosting exercise abilities.^[13]^

Nevertheless, there is still a lack of high-quality evidence to support the effectiveness of respiratory rehabilitation training on elderly patients with COVID-19. So this study will systematically evaluate the efficacy of this treatment and provide evidence-based guidance for clinical applications.

## 2 Materials and methods

This study will conduct its work by following the Preferred Reporting Items for Systematic Reviews and Meta-analysis Protocols (PRISMA-P) statement guidelines.^[14]^ And this study has been registered with PROSPERO at registration number: CRD42020199798.

### 2.1 Selection criteria

#### 2.1.1 Type of studies

Randomized controlled trials (RCTs) that explore the efficacy of respiratory rehabilitation training on elderly patients with COVID-19 will be included.

#### 2.1.2 Types of patients

Inclusion criteria:

(1) Patients with a definite diagnosis of COVID-19;
(2) Patients of 65 years old or above;
(3) Patients > 6 months after the onset of other acute diseases;
(4) Patients with a mini-mental state examination (MMSE) score > 21;
(5) Patients without chronic obstructive pulmonary disease (COPD) or other respiratory diseases; and
(6) Patients with a forced expiratory volume in 1 second (FEV_1_) > 70%.

Exclusion criteria:

(1) Patients with moderate or severe heart disease (Grade IIl or IV, New York Heart Association);
(2) Patients with severe ischemic or hemorrhagic stroke or neurodegenerative diseases.

#### 2.1.3 Types of interventions and comparisons

Treatment group: respiratory rehabilitation, with no limits to the number of courses and times. Control group: only receiving the standard care.

#### 2.1.4 Types of outcomes

Main outcomes:

(1) Respiratory function (FEV_1_);
(2) Forced vital capacity (FVC);
(3) The 6-minutes walk test (6MWT);
(4) Activities of daily living (ADL).

Additional outcome:

(1) Quality of life, anxiety, and depression scale scores.

### 2.2 Exclusion criteria

(1) Studies published repeatedly;
(2) Studies whose literature forms are abstracts or conference papers, with no ways to obtain the original data;
(3) Studies whose data are incomplete or have obvious errors that cannot be addressed after communications with the authors;
(4) Studies with wrong random methods.

### 2.3 Search strategy

The 2 researchers will independently conduct electronic searches on the following databases: Embase, MEDLINE, PubMed, Cochrane Library, China national knowledge infrastructure database, Wan Fang database, Chinese Science and Technology Periodical Database, and Chinese Biomedical Literature Database, with the retrieval period running from their inception to August 2020. A search strategy that combines MeSH terms and free words will be adopted. Taking PubMed as an example, the retrieval strategy is shown in Table 1.

**Table 1:**
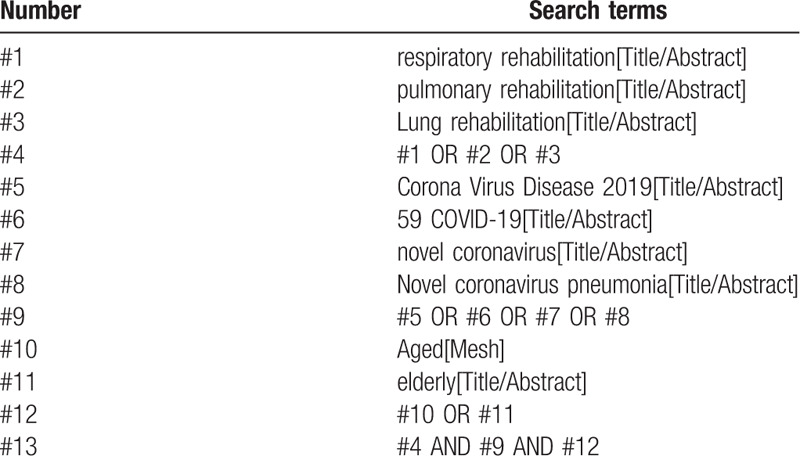
Search strategy in PubMed database.

### 2.4 Study selection and data extraction

#### 2.4.1 Selection of studies

**Figure 1:**
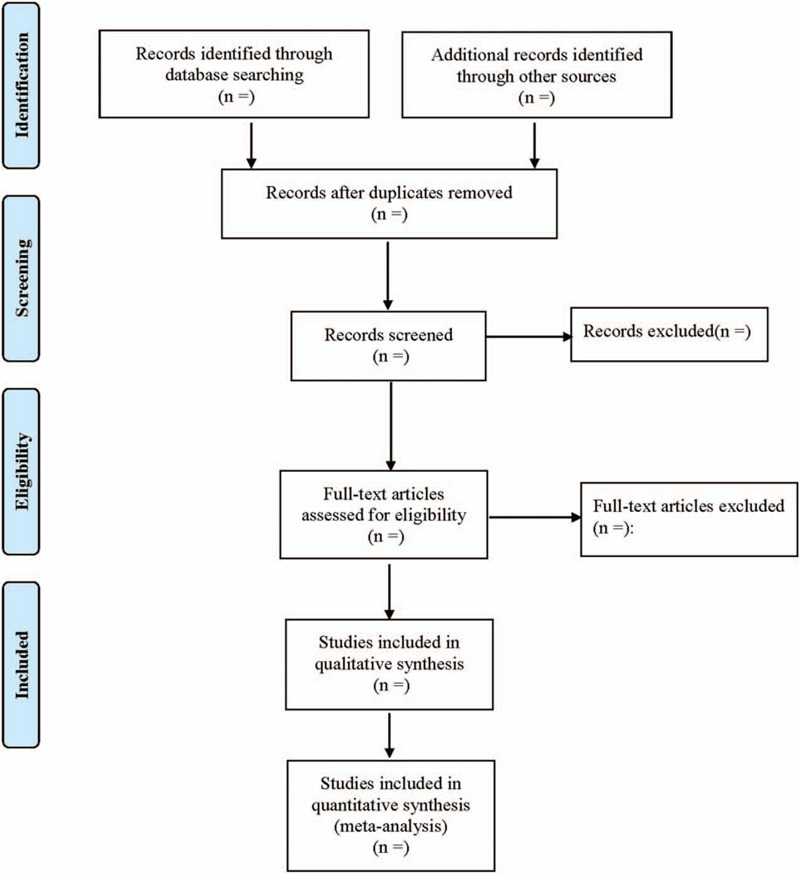
Flow diagram of study selection process.

The 2 reviewers will review topics and abstracts independently according to the research criteria and search strategies. If an article cannot be determined, the full text will be deleted. The excluded articles and the reasons for the exclusion will be recorded. Any objections will be settled through discussion with other reviewers. The detailed selection process is shown in Figure 1.

#### 2.4.2 Data extraction

Two independent reviewers will extract the data, including the publishing year, characteristics of participants, disease severity, sample size, age, study cycle, intervention details, results, adverse events, and others. For missing or unclear data, try to solve by discussing in the group and contacting the corresponding authors.

#### 2.4.3 Assessment of risk of bias

The Cochrane collaboration's tool for assessing risk of bias is used to assess the risk bias in the included studies. Two researchers determine the literatures from 3 levels, that is, low-risk, unclear, and high-risk, based on the performance of the included literatures in the above evaluation items. After completion, they would recheck. In case of a disagreement, they would discuss. If no agreement could be reached, a decision would be made in consultation with researchers from a third party.

#### 2.4.4 Measures of treatment effect

Standardized mean difference (SMD) is selected, and all the above data are represented by effect values at 95% confidence intervals (CIs).

#### 2.4.5 Dealing with missing data

The reviewers will try to obtain the missing data by contacting the corresponding authors. If failed, the data will be analyzed based on the existing information.

#### 2.4.6 Assessment of heterogeneity

*Q* test is used to qualitatively determine inter-study heterogeneity: if *P* ≥ .1, there is no inter-study heterogeneity; if *P* < .1, there is inter-study heterogeneity. At the same time, *I*^2^ value is used to quantitatively evaluate the inter-study heterogeneity: If *I*^2^ ≤ 50%, the heterogeneity is considered to be small; if *I*^2^ > 50%, significant heterogeneity exists.

#### 2.4.7 Assessment of reporting bias

For the major outcome indicators, if the number of the included studies is ≥10, funnel plot will be used to qualitatively detect the publication bias. Egger's and Begg's tests are used to quantitatively assess the potential publication bias.

#### 2.4.8 Data synthesis

If there is no statistical heterogeneity among the results, the fixed effect model will be used for meta-analysis. If there is statistical heterogeneity among the results, a random effect model will be used. If there is obvious clinical heterogeneity, subgroup analysis or sensitivity analysis will be performed. Meta-analysis will be conducted by using RevMan5.3 e (The Cochrane Collaboration, Oxford, England) and Stata 14.0 software (Stata Corporation, College Station, TX).

#### 2.4.9 Subgroup analysis

A subgroup analysis based on the intervention time (<6 weeks or ≥6 weeks) will be carried out.

#### 2.4.10 Sensitivity analysis

In order to test the stability of meta-analysis results of outcomes, a one-by-one elimination method will be adopted for sensitivity analysis.

#### 2.4.11 Ethics and dissemination

This systematic review will not require ethical approval, because no data used in this study are linked to individual patients’ information. The results will be disseminated only in a peer-reviewed publication.

## 3 Discussion

COVID-19 mainly damages the respiratory system, with strong infectivity and high fatality rate.^[15]^ The epidemic are seriously affecting the employment, life, and mental and physical health of residents, while disturbing the social and economic development.^[16]^ For COVID-19 patients, due to lung injuries, the lung functions will be weakened, and accordingly, the labor ability and exercise tolerance will also be impaired, thus seriously affecting the quality of life.^[17–20]^ For COVID-19 patients who have been cured and discharged from hospitals, the rehabilitation treatment in the later stages is very important.^[21,22]^

Respiratory rehabilitation therapy consists of a series of scientific and effective health promotion procedures.^[23,24]^ First of all, it can only be carried out after a standard rehabilitation evaluation of lung functions or systemic functions; therefore, personalized schemes are emphasized, with main contents including training of cardiopulmonary, aerobic, strength and daily functions, as well as some psychotherapy treatments.

As far as known, this study is the first systematic review and meta-analysis of the effects of respiratory rehabilitation training on elderly patients with COVID-19. Widely used for patients with chronic respiratory diseases, respiratory muscle rehabilitation training is a kind of non-drug treatment, which is safe and easy to learn and implement at a low cost. Very suitable for COVID-19 patients, respiratory rehabilitation is beneficial to alleviating the symptoms of pneumonia, boosting cardiopulmonary endurance, and improving physical and mental health, while enhancing patients’ ability to gradually recover and participate in social activities. Studies have shown that in the stable stage, the earlier rehabilitation intervention will lead to better effects.^[11]^

Due to limited original research discoveries, however, it is uncertain whether respiratory rehabilitation therapy can improve the pulmonary function of elderly patients with coronavirus pneumonia, a major concern for the medical community. It is noteworthy that the lack of sufficient RCT may be a limitation for this meta-analysis.

## Author contributions

**Data collection:** Lang Wang and Xiangjun Luo

**Funding support:** Yonghong Ouyang

**Literature retrieval:** Lang Wang and Xiangjun Luo

**Software operating:** Lang Wang and Xiangjun Luo

**Supervision:** Yonghong Ouyang and Qian Zhan

**Writing – original draft:** Huan Yan and Yonghong Ouyang

**Writing – review & editing:** Huan Yan and Yonghong Ouyang
